# Therapeutic potentials and challenges of cytostatic persister cancer cells

**DOI:** 10.18632/oncotarget.28488

**Published:** 2023-12-01

**Authors:** Paul Y. Kim, Cheuk T. Leung

**Keywords:** persister cancer cells, cytostatic therapy, cancer recurrence, PTEN/PI3K/AKT, proteasome inhibitor

Cancer cells that remain viable despite treatment constitute a persister condition that is implicated in residual diseases and a source from which resistant clones and relapses can emerge. Unlike resistant cells that are capable of cycling under therapy, persister cancer cells stay viable but assume a quiescent or non-proliferating state that is reversible upon treatment discontinuation. A source of persisters that has been under extensive study is drug-tolerant persisters, a small cancer cell population that can withstand the selection pressure of cytotoxic treatment and have been attributed to failure in achieving complete response. It is well recognized that many targeted therapeutic agents possess cytostatic effects that suppress growth without directly inducing cell death. While representing favorable responses, treatment-mediated cytostatic conditions require continual maintenance and intrinsically confer an obligate persister population throughout therapy. However, few efforts have focused on understanding the properties of such cytostatic persisters and exploring their therapeutic potentials.

A recent study [1] from our group explored the cellular controls in persister cancer cells under treatment-mediated cytostatic conditions and devised strategies for targeting to reduce cancer recurrence. Using a paradigm of maintenance cancer treatment with cytostatic targeted agents and cancer cells harboring genetic alterations predisposed to cancer recurrence [2–5], we observed that the persister cancer cells can sustain an elevated oncogenic AKT signaling while remaining in a non-proliferating state. Interestingly, given the role of AKT in cancer cell survival [6], the hyperactive AKT signaling is dispensable for survival in the cytostatic persisters [1]. This observation contrasts previous studies of drug-tolerant persisters under cytotoxic drug treatments, in which AKT signaling plays an important role for survival [7], and implicates a difference in cellular control between cytostatic and drug-tolerant persisters.

These initial findings prompted us to investigate the general effects of oncogenic AKT signaling on non-proliferating cells. To do that, we undertook a bottom-up approach and devised a platform amendable for engineering cell signaling in cytostatic states using three-dimensional culture of quiescent mammary organoids. Quantitative proteomic analysis showed that hyperactive AKT signaling alters the homeostatic control in cytostatic state and promotes an oxidative and proteotoxic environment. Screening a small set of pharmacologic agents that further perturb redox and proteostasis identified an efficacy of proteasome inhibitors in inducing apoptosis in the cytostatic persister cancer cells with selectivity over quiescent normal cells. Mechanistically, this induction of apoptosis is dependent on p70S6K, redox, and ASK1 [1]. These results underscore an altered homeostatic control driven by sustained oncogenic signaling in non-proliferating state that is exploitable for synthetic-lethal targeting.

Notably, this targeting approach shows efficacy in cytostatic persisters harboring PTEN/PI3K pathway mutations independent of breast cancer subtypes (ER^+^, Her2^+^, and triple negative), epithelial tissue origins (breast, lung and ovarian), and drug-mediated cytostatic conditions (CDK4/6 and EGFR/Her2 inhibitors). Furthermore, tumor spheroids and xenograft models of lung and breast cancer showed that targeting cytostatic persisters could significantly reduce and delay cancer recurrence after treatment discontinuation.

Findings from this study have shed light into the cellular controls in cytostatic persisters and highlighted that treatment-mediated cytostatic condition before resistance emerges is a viable targeting venue to reduce cancer recurrence. This study also raised insights into the complexity and challenges of targeting persister cancer cells in therapy. Investigating oncogenic AKT signaling under cytostatic conditions has suggested a different homeostatic environment between cytostatic and drug-tolerant persisters despite similar non-proliferating status, which may contribute to the distinct approaches for targeting. Inhibiting the pro-survival oncogenic AKT signals have broad efficacy in targeting drug-tolerant persisters [7, 8]. We speculate that the viability of drug-tolerant persisters is more reliant on active oncogenic survival signals because the cells are generally under continuous cytotoxic selection pressure. In contrast, cytostatic persisters might be subjected to treatment-mediated growth suppression without direct selection pressure and, therefore, are less reliant on active oncogenic signals for survival. Rather, sustained active oncogenic signals, such as those caused by existing genetic mutations in the persisters, could affect the homeostatic environment and confer an exploitable vulnerability for synthetic-lethal targeting while remaining in the treatment-mediated cytostatic state.

The synthetic-lethal approach for targeting is dependent on the effects of specific oncogenic signaling on the cytostatic state homeostasis, suggesting that distinct approaches may be necessary for targeting cytostatic persisters harboring different oncogenic alterations. This notion emphasizes a value of identifying sustained oncogenic signals and their effects in cytostatic persisters, which could facilitate the development of targeting strategies. More broadly, given the pharmacokinetic limitations and the dose-dependent cytostatic and cytotoxic effects of many cancer therapeutic agents, we anticipate that heterogeneous persister populations are inevitable in treatment. The distinct vulnerabilities of cytostatic and drug-tolerant persisters imply that administering multiple targeted regimens would be necessary to effectively deplete the persister reservoirs in patients under cancer treatments.
